# Activation of the ATX-LPA axis by carcinogenic chemicals: New leads to prevent pancreatic cancer? 

**DOI:** 10.17179/excli2020-1192

**Published:** 2020-04-09

**Authors:** Johan Högberg

**Affiliations:** 1 Karolinska Institutet, Institute of Environmental Medicine, C6 Biochemical toxicology, 17177 Stockholm, Sweden

## ⁯

***Dear Editor,***

A recent article (Auciello et al., 2019[1]) is of considerable interest for understanding the tumor biology of pancreatic ductal adenocarcinoma (PDAC). In addition, it may increase our possibilities to find new strategies for preventing this deadly cancer type. 

The Auciello study connects PDAC with the secreted inflammatory protein autotaxin (ATX). The study describes a metabolic shift of lipids in pancreatic tumor tissue, with increased lipid production in stromal cells that might nurture cancer cells in a paracrine fashion. Of specific interest is the increased release of lysophosphatidylcholeine (LPC) from stromal cells, interplaying with the activated secretion of ATX from PDAC cells. ATX is a lipase that produces lysophosphatidic acid (LPA), a signaling molecule previously known to stimulate proliferation and migration of cancer cells. The study by Auciello shows that the LPC-ATX-LPA axis stimulates growth and progression of implanted genetically modified PDAC cells in mice. Although convincingly thorough in many details, the article does not answer questions about the upstream events that lead to this ATX activation. 

Seven years ago, we published a study (Kadekar et al., 2012[5]) that might give clues to this question. We analyzed chemicals tested by NTP in 2-year cancer bioassays. We focused on the relatively few chemicals that significantly increased exocrine pancreatic tumors in male rats and included eight substances. We asked the question if these chemicals had carcinogenic effects in common. To our surprise we were able to identify ATX and showed that all eight chemicals triggered ATX expression and stimulated ATX secretion in cultured human PDAC cells. We concluded that ATX is a target for xenobiotic chemicals that may promote pancreatic tumor development. Incidentally, we also found that testosterone potentiated this effect, an effect that might explain why PDAC more often affects men than women. A strength of our study is that we started with an unbiased literature analysis by using an automated text mining tool (http://crab3.lionproject.net/). Another strength is that we based the selection of study chemicals on well documented and strictly controlled standardized 2-year cancer bioassays.

Data in Auciello et al. (2019[1]) thus confirm our seven-year old study by strengthening a connection between ATX and pancreatic exocrine cancer. Of importance here is that our study also implicates carcinogenic chemicals. Whether these pancreatic tumorigenic chemicals primarily targeted pancreatic tissue in the NTP assays is not known. However, it can be anticipated that the LPC-ATX-LPA axis is of special importance in this organ, as the altered lipid metabolism might not only provide aberrant growth stimuli via the LPC-ATX-LPA axis, but also by providing nutrients for tumor growth (Auciello et al., 2019[1]). To summarize, our study suggests that the ATX activation, as described by Auciello, can be a response to xenobiotic chemicals. The tumorigenic effect of these chemicals, as documented in the NTP bioassays, suggests that this activation might be an early, or even initiating, driving force in PDAC development.

In the literature, high ATX activity is associated with inflammation, fibrosis and cancer (Zheng et al., 2017[7]). ATX is rarely described as a target for xenobiotics. We did so in Kadekar et al. (2012[5]), and included more toxic substances in later studies. For example, we have evidence indicating that ATX might be a primary target for some notorious toxicants such as diisocyanates (Brostrom et al., 2015[4], 2018[3]) and crystalline silica (Zheng et al., 2017[7]). We also have data from occupationally exposed humans and from cell experiments suggesting that low concentrations of these agents (lower than current occupational exposure limits) rapidly stimulate ATX secretion from bronchial epithelial cells (Brostrom et al., 2015[4], 2018[3]).

PDAC is a deadly disease with few 5-year survivors, and there are no indications of recent improvements in 5-year survival statistics. Thus, there is an urgent need for new preventive strategies. There are few environmental exposures associated with PDAC, but our study (Kadekar et al., 2012[5]) in combination with the Auciello study (Auciello et al., 2019[1]) provide leads for establishing new chemical risk factors. Additional experimental studies should characterize the role of ATX-activating chemicals in PDAC development and ATX-activating chemicals should be investigated in epidemiological studies. Interestingly, a simple literature search on PubMed shows that there are at least two independent epidemiological studies (Birk et al., 2009[2]; Kauppinen et al., 1995[6]) documenting significant associations between PDAC and occupational exposure to crystalline silica. 

## Conflict of interest

The author declares no conflict of interest.

## Funding

This work was financially supported by Forte, Sweden (proj. nr 2018-00610). 
